# Are Antibody Panels Under‐Utilized in Movement Disorders Diagnosis? Yes

**DOI:** 10.1002/mdc3.13171

**Published:** 2021-03-25

**Authors:** Bettina Balint

**Affiliations:** ^1^ Department of Neurology University Hospital Heidelberg Heidelberg Germany

**Keywords:** antibodies, diagnosis, movement disorders, testing, treatment

## Autoimmune Movement Disorders—Rare, Why Should I Care?

The last decade has seen a revolution in neuroimmunology, with a rising number of new antibodies defining autoimmune neurological disease, including movement disorders. Yet, still several reports indicate a high rate of missed or delayed diagnoses in patients with autoimmune encephalitis: in one study, nearly 40% of such patients were suspected to have prion disease^1^; in another study, the correct diagnosis of autoimmune encephalitis was initially only considered in 32%, while the other 68% received alternate diagnoses like normal pressure hydrocephalus, dementia with lewy bodies or functional neurological disorder.^2^ If presentations were atypical for classic autoimmune encephalitis, the correct diagnosis was only suspected in 2%.^2^

The low index of suspicion may arise from the fact that autoimmune encephalitis generally is considered rare. However, it's incidence and prevalence are similar, or surpass in certain subgroups, that of infectious encephalitis.^3^, ^4^ Moreover, with the wider use of immune checkpoint inhibitors, we will likely see an increasing frequency of autoimmune neurological disease, including movement disorders.^5^

In contrast, however, to the many neurodegenerative or genetic movement disorders for which the future will hopefully hold disease‐modifying therapies, autoimmune movement disorders are already treatable now. A timely diagnosis is crucial, because the earlier the treatment, the better the outcome.^6^, ^7^

## Aren't Clinical Features, MRI and CSF Enough to Diagnose Autoimmune Encephalitis?

To avoid delays in treatment due to waiting for antibody test results, an international expert panel suggested criteria for possible autoimmune encephalitis, based on clinical features, MRI and CSF.^8^ These include: rapid onset (<3 m); either new focal CNS findings, seizures, CSF pleocytosis or MRI abnormalities; and reasonable exclusion of other causes.

However, subsequent studies showed that a proportion of patients will be missed with this approach. For example, 13% of patients with anti‐LGI1 encephalitis did not meet the criteria, nor did 15% of a cohort of mixed autoimmune encephalitides, because those clinical and paraclinical criteria were not sensitive enough.^2^, ^9^

Indeed, while we think of encephalitis as a disease with rapid onset, some antibodies associate with an insidious disease course, even mimicking degenerative disease, for example, those against LGI1, DPPX, CASPR2, IgLON5.^9^, ^10^, ^11^ Some antibodies have a broad phenotypic spectrum and can present with unusual phenomenology, such as NMDAR, GABA_A_R, IgLON5 or CASPR2 antibodies.^12^, ^13^, ^14^, ^15^ Particularly such cases with “atypical” movement disorder presentations are at a high risk of misdiagnosis.^2^

A meta‐analysis showed that the MRI in autoimmune encephalitis is often normal or unspecific.^16^ CSF findings differ across antibody subtypes, but even where pleocytosis, oligoclonal band or protein elevation are relatively frequent, 40%, 50% and 30% of cases will feature normal results for these markers, respectively.^17^ For example, patients with LGI1 antibodies may have a normal MRI and CSF, the reason why Graus and colleagues coined the term LGI1 encephalopathy (rather than encephalitis).

In summary, CSF and MRI remain important investigations in the diagnostic work up of such cases. The introduction of clinical criteria with widely available diagnostic tools like CSF and MRI was an important contribution to avoid any delay in treatment, but they have limitations regarding their sensitivity, and there remains a significant proportion of patients requiring specific antibody testing to make the diagnosis.

Besides, one movement disorder phenotype can occur with various antibodies.^18^ For example, in stiff person spectrum disorder (SPSD), the phenotype does not allow accurate prediction of the underlying antibody, which may be anti‐GAD, GlyR, amphiphysin or DPPX, with differing implications.^19^ Autoimmune parkinsonism may be seen with CRMP5, Ma2, Ri, LGI1, DPPX or IgLON5 antibodies. Same applies for other movement disorders, with cerebellar ataxia being the extreme example with approximately 30 different antibodies – with different further implications.

## Beyond the Phenotype: Antibodies Indicate Relevant Disease beside the Autoimmune Neurological Syndrome

Apart from the formal diagnosis of an autoimmune (movement) disorder, knowing the specific underlying antibody is important as it can indicate relevant associated illnesses, in particular tumors or other, organ‐specific autoimmunity.

Antibodies may be a paraneoplastic phenomenon, indicative of malignancies, and the paraneoplastic syndrome may precede cancer diagnosis by many years.

**FIG. 1:**
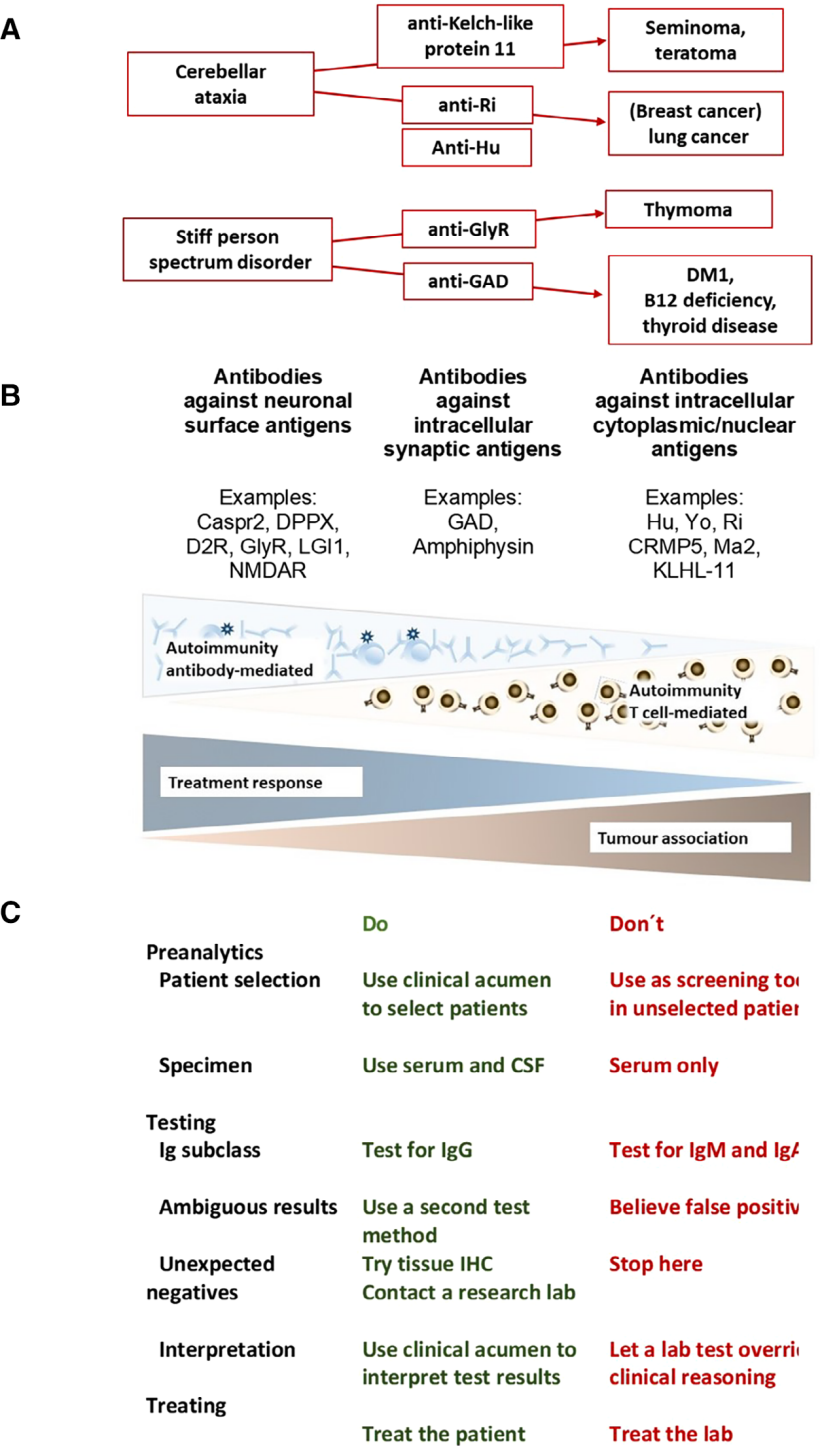
(A) One phenotype, many antibodies with different implications. In a patient with cerebellar ataxia with Kelch‐like protein 11 antibodies, one needs to screen first and foremost for seminomas in men, or teratomas in women; in a woman with Ri antibodies, one would suspect primarily breast cancer, and a little less likely, lung cancer; however, if the patient has Ri and also Hu antibodies, such combination would indicate lung cancer. (B) Three groups of neuronal antibodies and their pathogenic roles, examples, treatment responses and tumor associations (adapted from^18^). (C) The dos and Don'ts of antibody testing.

Onconeuronal antibodies are an important part of the diagnostic criteria for paraneoplastic syndromes and have a high specificity if tested properly.^20^, ^21^, ^22^ Moreover, because of their strong association with particular types of cancer, they allow orientating the tumor search at a stage where it is frequently not clinically overt, and justify surveillance in patients where the cancer is not detectable as yet.^21^, ^23^, ^24^ For example, in a patient with cerebellar ataxia the tumor screening can be tailored according to the identified antibodies: with Kelch‐like protein 11 antibodies, one needs to screen first and foremost for seminomas in men, or teratomas in women^25^; in a woman with Ri antibodies, one would suspect primarily breast cancer, and a little less likely, lung cancer; however, if the patient had not only Ri but also Hu antibodies, such combination would indicate a lung cancer (see Fig 1A).^24^ Another very illustrative case is that of a 31‐year old woman with a 3‐year history of slowly progressive cerebellar ataxia; while her CSF was normal (incl. no OCBs), her MRI showed a hot‐cross bun as seen in degenerative conditions like multisystem atrophy or spinocerebellar ataxia. Because of high titres of ITPR1 antibodies in serum and CSF, she was closely followed up, and 11 years after onset of the ataxia, a clinically non‐manifest breast cancer (expressing ITPR1) was detected.^23^ Not only onconeuronal antibodies, but also neuronal surface antibodies may point to an underlying malignancy (for example: NMDAR antibodies to teratoma, in up to ~40%, CASPR2 antibodies, to thymoma in ~20%–50%; DPPX antibodies to B cell malignancies in up to ~10%; GlyR antibodies to thymoma in ~10%; DNER antibodies to Hodgkin's lymphoma in ~90%).^18^

On the other hand, some antibodies strongly associated with other, organ‐specific autoimmune disorders. For example in patients with GAD antibodies, comorbidities like type 1 diabetes, thyroiditis or pernicious anemia are frequent (~50%, 20% and 7%, respectively).^26^

Accordingly, a SPSD patient with amphiphysin antibodies will be screened for breast cancer or small cell lung cancer; a SPSD patient with GlyR antibodies will be screened for a thymoma, and has an excellent prognosis once this is removed; while the patient with GAD antibodies will be regularly screened for the occurrence of diabetes, autoimmune thyroid disease or pernicious anemia.^19^ In a case of late onset chorea, Hu or CRMP5 antibodies warrant a tumor search, while IgLON5 antibodies are unlikely to occur with a malignancy; if LGI1 and CASPR2 antibodies are present, monitoring for potentially fatal cardiac arrhythmias needs to be considered, illustrating that associated relevant illness extends beyond malignancies or wider autoimmunity, but includes also specific antibody effects.^27^, ^28^

## Antibodies Inform Prognosis and Guide Treatment

If the underlying immunobiology of an autoimmune movement disorder is defined by an identified antibody, it informs the prognosis and guides the therapeutic strategy and, of practical importance, alleviates approval of expensive immunotherapy funding by health insurances.

Generally, we recognize three groups of antibodies (see Fig 1B) – those against intracellular antigens, the classic onconeuronal antibodies (like Hu Yo Ri, Kelch‐like protein 11); the neuronal surface antibodies (eg, against NMDAR, LGI1, DPPX, IgLON5), and the intermediate group with intracellular, but synaptic antigens like GAD and amphiphysin—and we know that these groups vary in treatment response and prognosis because of differing underlying immunobiology. For example, a patient with late‐onset chorea with Hu or CRMP5 antibodies has very likely a malignancy and treatment aims at preventing a worsening, but is unlikely to reverse the symptoms, given the neuronal damage is mediated by cytotoxic T cells and irreversible. In contrast, late‐onset chorea with surface antibodies like LGI1 or CASPR2 has a much better prognosis. Even within these antibody groups, there are differences because of the specific pathophysiological mechanisms of antibodies (reversible vs. irreversible downregulation of the target, complement activation etc.) and risk of relapses, resulting in different treatment and monitoring strategies.^29^ For example, the downregulation of NMDAR by antibodies is reversible, and patients tend to improve significantly with early, appropriate treatment. Half of the patients need second line immunotherapy (eg, rituximab, cyclophosphamide). Overall, there is a 2‐year case fatality rate of ~12% and a ~ 12% chance of one or more relapses in the 2 years after onset.^6^, ^30^ Patients with CASPR2 antibodies have a higher risk of relapses (~25% in up to 7 years after initial presentation), have similar 2‐year case fatality rate (~10%) but have a slightly worse overall outcome (stabilization or improvement in 40%).^30^, ^31^ Patients experience relapses in approximately a third of cases relapses in 8 years after onset, with a 2‐year case fatality rate of approximately 20%, but overall, first line therapy seems to suffice. Of note, 66% of cases will suffer from persistent cognitive deficits that will not improve with escalated treatment.^9^, ^32^ Anti‐IgLON5 disease portends a higher mortality (~34%) due to sudden death and aspiration, and an escalated/combination immunotherapy, for example, with steroids, optionally in combination with steroid‐sparing agents, seems to be necessary to achieve sustained stabilization or improvement.^33^, ^34^

Beside the target of antibodies, further definition of the exact immunopathophysiology can inform our therapeutic approach. For example, in disease with predominantly compliment activating IgG1 antibodies (eg, AQP4), Eculizumab is a promising option, while it less likely to benefit those with a mainly IgG4 driven disease (eg, IgLON5). Thus, with trials investigating specific treatments in molecularly defined disease and a better understanding of the underlying immunology, recommendations for treatment will become more specific for each disease in the future.^35^

## Antibody Panels as Diagnostic Test—The Future Is Now

There are many good reasons, as outlined above, for a molecular diagnosis of autoimmune movement disorders, in particular securing the diagnosis and tailoring the treatment. There are also many practical reasons in favor of antibody testing: it requires only serum and CSF, both routinely obtained, and yields fairly fast results. Considering the costs and health‐economic implications of longer hospitalization, increased morbidity and mortality in patients that are not correctly and quickly diagnosed with an autoimmune movement disorder, the cost–benefit ratio also favors antibody testing. Costs vary from country to country; in Europe, the price of a single antibody is ~39€, that of a biochip mosaic panel with many antibodies ~240€. In some countries, of course, costs are still a hindrance but there are enough examples how such obstacle have been mastered with collaborative efforts with research labs or establishment of own laboratories.^36^, ^37^

Akin to the developments in the field of genetics, where gene panels have become widely available in a fairly short time span, same will apply to antibody panels, and costs will similarly decrease with wider application and technological advances.

## A Word of Caution and a Guide to Antibody Testing

Given that it is often difficult to infer the underlying antibody just by the phenotype, phenotype‐based panels are preferable over single antibody testing,^38^ and there are also tools to design a panel based on a particular patient's phenotype.^18^

The sensitivity and specificity of most neuronal antibodies for neurological autoimmune disease is well established.^39^ In contrast, there are still controversial antibodies of limited clinical relevance, like “basal ganglia antibodies”^40^ or the “Cunningham panel”^41^ (not targeting conformational epitopes in vivo) that are not recommended for routine testing and not subject of this article.

Some confusion arose from publications with astonishingly high seroprevalence rates of neuronal antibodies in healthy people and patients with other, not primarily autoimmune diseases.^42^ To avoid such ambiguous or “false positive” (in the sense of not indicating neuroimmunological disease) test results, the Dos and Don'ts of antibody testing (see Fig. 1C) should be considered. The current recommendation is to perform antibody testing based on clinical or paraclinical features suggestive of an autoimmune disorder, not as a screening procedure. It is important to test serum and CSF (apart from very few exceptions) to achieve highest sensitivity and specificity, rather than serum only which may yield ambiguous results.^42^ Similarly, in some publications, testing for IgM and IgA was included, though evidence of IgM or IgA antibodies being pathogenetically or diagnostically relevant is lacking.^43^ Confirmation of ambiguous test results with a second, different test method – for example, a cell‐based assay and tissue immunohistochemistry—is good practise and would probably have dispelled many of the reported positive test results in subjects with no autoimmune neurological disease. Another pitfall are negative test results where there is a strong suspicion of an autoimmune disease—this may occur, for example, with a new antibody, the target of which is not yet identified, where tissue immunohistochemistry or contacting a research lab may be the way forward. Just as clinical acumen has to guide the interpretation of test results or the decision to pursue further testing, it is also of paramount importance when it comes to treatment. Current evidence does not support basing for example the escalation or continuation of treatment on an antibody test result. For example, LGI1 antibodies can be detected up to 2 years after full recovery.^9^

## Conclusions

Neuronal antibody panels are a very useful diagnostic tool and have clinically relevant implications for disease monitoring, treatment and prognosis. Like genetic panels, antibody panels will become more widely available soon and, used properly, improve timely identification and management of autoimmune movement disorders.

## Author Roles

(1) Research project: A. Conception, B. Organization, C. Execution; (2) Statistical Analysis: A. Design, B. Execution, C. Review and Critique; (3) Manuscript Preparation: A. Writing of the first draft, B. Review and Critique.

B.B.: 3A, 3B

## Disclosures

### Funding Sources & Conflicts of Interest

No specific funding was received for this work, and the author has no conflicts of interest relevant to this work.

### Financial Disclosures for Previous 12 Months

BB reports no disclosures.

### Ethical Compliance Statement

The author confirms that neither patient consent nor the approval of an institutional review board was required for this work. We confirm that we have read the Journal's position on issues involved in ethical publication and affirm that this work is consistent with those guidelines.
